# The Molecular and Cellular Mechanisms of Endometriosis: From Basic Pathophysiology to Clinical Implications

**DOI:** 10.3390/ijms26062458

**Published:** 2025-03-10

**Authors:** Heidi Mariadas, Jie-Hong Chen, Kuo-Hu Chen

**Affiliations:** 1 Department of Obstetrics and Gynecology, Taipei Tzu-Chi Hospital, The Buddhist Tzu-Chi Medical Foundation, New Taipei City 23142, Taiwan; heidimariadas@gmail.com; 2 Department of Medicine, MacKay Medical College, New Taipei City 25245, Taiwan; albertjhc@gmail.com; 3 School of Medicine, Tzu-Chi University, Hualien 97004, Taiwan

**Keywords:** endometriosis, endometrial mesenchymal stem cells, natural killer cell

## Abstract

Endometriosis is a complex gynecological disorder characterized by endometrial-like tissue growing outside the uterus, leading to chronic pain, infertility, and reduced quality of life. Its pathophysiology involves genetic, epigenetic, immune, and molecular factors. Theories such as retrograde menstruation, coelomic metaplasia, and stem cell involvement explain lesion formation. Endometrial mesenchymal stem cells (eMSCs) and epithelial progenitors (eEPs) contribute to lesion establishment by adhering to peritoneal surfaces, proliferating, and differentiating into ectopic tissue. Aberrant adhesion molecules, inflammatory cytokines, and molecular pathways like PI3K/Akt and Wnt/β-catenin drive proliferation, angiogenesis, and resistance to apoptosis. Elevated estrogen levels and progesterone resistance further promote lesion growth and immune evasion. Immune dysfunction, including altered macrophage activity and reduced natural killer (NK) cell function, contributes to inflammation and lesion persistence. Pain is linked to prostaglandin E2 (PGE2) and nerve infiltration, emphasizing the need for targeted pain management. Current therapies, such as GnRH agonists, suppress ovarian hormone production but face limitations in long-term efficacy and side effects. Integrating molecular insights into clinical practice may advance diagnostics and treatment, with emerging approaches focusing on molecular pathways, immune modulation, and hormonal regulation for more effective, personalized therapies. Future research should unravel the complex mechanisms driving endometriosis to improve patient outcomes.

## 1. Introduction

Endometriosis is a complex, chronic condition characterized by the presence of endometrial-like tissue outside the uterus, which induces a long-lasting inflammatory response. The exact pathophysiology of endometriosis remains unclear, and it is likely multifactorial, involving genetic, immunologic, hormonal, and environmental factors. Nonetheless, several theories and types of pathophysiological mechanisms have been proposed to explain the development and progression of endometriosis. Understanding the pathophysiology of endometriosis is crucial for developing effective treatments and management strategies.

Endometriosis is classified into four stages based on the Revised American Society for Reproductive Medicine (rASRM) classification, which evaluates the location, extent, and depth of lesions, as well as the presence of adhesions and ovarian endometriomas. Stage I (Minimal) is characterized by a few small superficial lesions with minimal or no adhesions. Stage II (Mild) involves slightly deeper implants and mild adhesions, often with scattered lesions on the peritoneum and ovaries. Stage III (Moderate) includes multiple deep implants, small ovarian endometriomas (chocolate cysts), and dense adhesions affecting the ovaries and fallopian tubes. Stage IV (Severe) is the most advanced form, with large ovarian endometriomas, extensive adhesions, and deep infiltrating lesions affecting pelvic organs such as the rectum, bladder, and intestines. However, the severity of symptoms does not always correlate with the rASRM stage, as some women with minimal disease experience severe pain, while others with extensive lesions remain asymptomatic [1,2].

Several theories attempt to explain the mechanisms of endometriosis development. Retrograde menstruation, the most widely known, suggests the spread of endometrial cells to ectopic sites but fails to explain cases in non-menstruating individuals. The vascular and lymphatic dissemination theory supports systemic spread, while coelomic metaplasia suggests transformation of peritoneal cells into endometrial-like tissue. Genetic and epigenetic factors further influence disease susceptibility and progression.

Endometrial stem cells (endometrial mesenchymal stem cells [eMSCs] and endometrial epithelial progenitors [eEPs]) play key roles in lesion establishment, possessing strong adhesive and proliferative properties. Altered integrin and cadherin expression enhances adhesion to peritoneal surfaces, while inflammation sustains lesion growth. Additionally, endometrial stromal cells (ESCs) and smooth muscle cells (SMCs) contribute to lesion heterogeneity, originating from basal endometrial layers or reactivated coelomic epithelium [1,2,3].

Molecular dysregulation underlies disease progression, with aberrant adhesion, invasion, and apoptosis resistance. Elevated estradiol and progesterone resistance, driven by epigenetic modifications, promote lesion survival. Dysregulated pathways—PI3K/Akt, Wnt/β-catenin, and JAK/STAT—fuel proliferation, immune evasion, and angiogenesis. Recurrent lesion bleeding triggers chronic inflammation, adhesion formation, and pain [2,3].

Inflammation is central to endometriosis, with immune dysfunction exacerbating disease severity. Peritoneal macrophages produce excessive cytokines (TNF-α, IL-1β, and IL-6), while diminished NK cell activity allows ectopic cells to evade immune clearance. NF-κB overactivation and ROS generation further damage tissues, reinforcing chronic pain and inflammation [2,3].

Currently, targeted therapies are emerging, aiming to modulate hormonal and inflammatory pathways. GnRH agonists and antagonists suppress estrogen, reducing lesion growth. Novel treatments focus on inhibiting the pathways of PI3K/Akt, JAK/STAT, prostaglandins, and cytokines. Anti-angiogenic strategies targeting VEGF, TGF-β, and IGF offer promising avenues for treatment [4]. This review summarizes current advances in the research of endometriosis pathophysiology, molecular and cellular mechanisms, as well as treatment. As research progresses, a more comprehensive picture of the disease’s complex nature is emerging, paving the way for innovative therapeutic approaches.

## 2. Methods

The literature was searched to retrieve basic and clinical research that investigated the pathophysiology of endometriosis, along with molecular and cellular mechanisms, and further clinical implications, especially in the field of pain and treatment. In this review, all studies were solicited from the databases PubMed and Ovid Medline using the search terms “endometriosis”, “pathophysiology”, “molecular mechanism”, “cellular mechanism”, and “clinical implication” for the research topic. For screening and selection in the second stage, only full-text articles written in English were considered for inclusion in a subsequent analysis. In the next stage, non-human-based research and duplicated articles were excluded.

**Figure 1:**
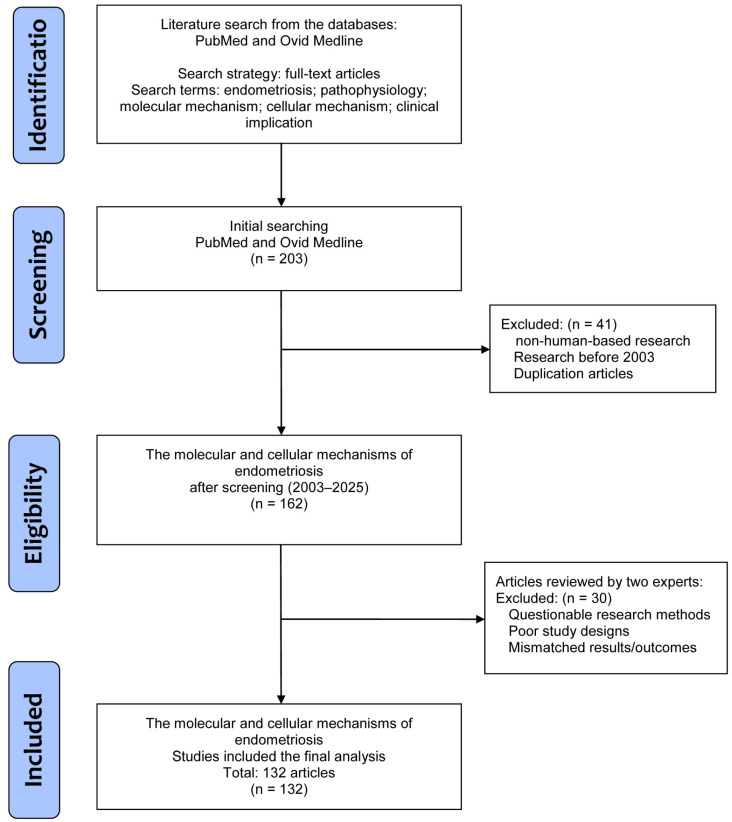
A flowchart of the database search, screening, selection, and inclusion of eligible articles from the literature.

Articles published before 2003 were also excluded to ensure novelty of this review by including all potential articles only in the last 20 years. Hereafter, two experts inspected the articles to exclude studies with poorer study designs, questionable methods, or unclear research outcomes. Finally, a total of 132 articles were eligible for selection in the current review (Figure 1).

## 3. Types of Endometrioses

Endometriosis manifests in different forms, with three main variants of lesions: superficial peritoneal disease (SPD), deep infiltrating endometriosis (DIE), and ovarian endometriomas [1,2,3,4].

### 3.1. Superficial Peritoneal Disease (SPD)

Superficial peritoneal disease is the most common and mildest form, where endometrial-like lesions are confined to the surface of the peritoneum. These lesions may appear as red, white, or pigmented spots and are typically associated with mild pelvic pain, especially during menstruation [2]. Diagnosis is often made through laparoscopy, since imaging may not reliably detect superficial lesions. SPD is less likely to cause significant fertility problems compared to other forms of endometriosis.

### 3.2. Deep Infiltrating Endometriosis (DIE)

In contrast, deep infiltrating endometriosis (DIE) is a more severe form where endometrial-like tissue infiltrates deeper into the pelvic organs, such as the rectum, bladder, and uterosacral ligaments, with a depth of penetration exceeding 5 mm [2,5]. This variant is known for causing chronic, debilitating pain, including severe dysmenorrhea, dyspareunia, and symptoms related to bowel or urinary function. DIE is often diagnosed using advanced imaging techniques like MRI or transvaginal ultrasound, and laparoscopy is used for staging. This form of endometriosis can cause significant complications, including bowel obstructions, and is more likely to affect fertility.

### 3.3. Ovarian Endometriomas

The third variant, ovarian endometriomas, refers to cysts on the ovaries or cyst filled with old blood (often called “chocolate cysts”) that develop when endometrial tissue grows on the ovaries [2]. These cysts are typically associated with pelvic pain, particularly during menstruation, and may lead to infertility due to their impact on ovarian function. Ovarian endometriomas can be detected with transvaginal ultrasound or MRI, with characteristic appearances of a ground-glass echotexture. Large cysts may require surgical removal, especially if they cause symptoms or hinder fertility. This variant has a high association with infertility and often requires specific management strategies to preserve ovarian function.

## 4. The Pathophysiology of Endometriosis

**Figure 2:**
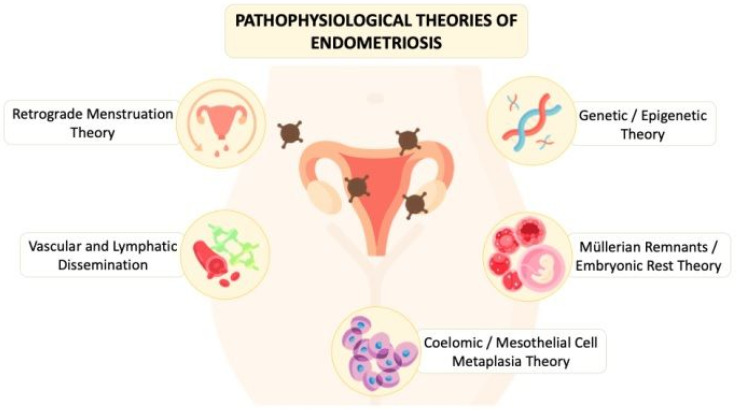
The diagram illustrates the pathophysiological theories of endometriosis proposed in the literature from the past to present.

The underlying pathophysiology of endometriosis involves complex interactions between cells, tissues, and organs and is not fully explored. As shown in Figure 2, the proposed theories that have been investigated by scholars include retrograde menstruation theory/endometriotic disease theory (EDT), vascular and lymphatic dissemination, coelomic metaplasia/mesothelial cell metaplasia theory, Müllerian remnants theories/embryonic rest theory, and genetic/epigenetic theory. However, none of the theories can solely explain all gross and microscopic changes found in a patient with endometriosis [1,2].

### 4.1. Retrograde Menstruation Theory/Endometriotic Disease Theory (EDT)

Retrograde menstruation theory is the most well-known theory, proposed by John Sampson in the 1920s, suggesting that during menstruation, endometrial cells flow backwards through the fallopian tubes into the ovaries and peritoneal cavity, which could lead to ovarian endometriomas and pelvic endometriosis, respectively [2,6,7]. Also, the retrograde menstrual blood containing endometrial cells and glands can invade and reside within the myometrium, resulting in adenomyosis. This theory is supported by higher incidences of the condition in women with genital tract obstructions. In cases of retrograde menstruation, these cells then seed on the pelvic organs and surfaces of pelvic walls or even infiltrate into deeper sites of the pelvis to potentially cause pelvic organ damage (endometriotic disease) or pain [8]. The most common sites of pelvic lesions include the ovaries, fallopian tubes, uterus, pelvic peritoneum, and uterosacral ligaments (USLs) [9]. However, since not all individuals with retrograde menstruation develop endometriosis, other factors should play a role [6,9]. Moreover, the theory does not account for the women who are found to have endometriosis in more distant sites or for those who do not menstruate (rare cases of extragonadal endometriosis and endometriosis in male patients) [6,10].

### 4.2. Vascular and Lymphatic Dissemination

Another theory by Sampson for the occurrence of endometriosis is vascular and lymphatic dissemination, which proposes that circulating blood cells originating from bone marrow or exfoliated endometrial cells differentiate into endometriotic tissues. The endometrial stromal cells (CD10) could spread to distant sites via blood vessels or lymphatics, leading to endometriosis of distal organs such as the lungs, liver, or brain, as another origin of extra-pelvic endometriosis [6,9,11,12].

### 4.3. Coelomic Metaplasia/Mesothelial Cell Metaplasia Theory

The hypothesis of coelomic metaplasia or the theory of mesothelial cell metaplasia, originally proposed in 1942 by Meyer, explains the occurrence of endometriosis in locations not accessible by retrograde menstruation and in the absence of a functioning uterus among Müllerian duct defects women, postmenopausal women, or cisgender men [2]. It suggests that various types of cells including peritoneal stem cells, peritoneal mesothelial cells, endometrial stem cells, bone marrow cells, and others can undergo metaplastic changes under the influence of hormonal or immune factors to transform into endometriotic cells to contribute to the development of diseases like endometriosis in the ovaries, fallopian tube, pelvic wall and abdomen, and cavities [6,7,12,13]. This theory is supported by evidence of mesothelial–mesenchymal transitions and the role of bone marrow cells in peritoneal repair, highlighting that the metaplastic cells involved in endometriosis are genetically and epigenetically similar to endometrial cells, aligning with theories of retrograde menstruation and implantation [13,14].

### 4.4. Müllerian Remnants Theories/Embryonic Rest Theory

In premenarcheal or rare cases of extra-pelvic endometriosis, the Müllerian remnants theories/embryonic rest theory suggests that endometriosis arises from vestiges of embryonic tissue (Müllerian, mesothelium, and bone marrow stem cell remnants) that can differentiate into endometrial tissue. Under certain stimuli, such as maternal hormone exposure or hormonal changes in puberty, remnant Müllerian cells can undergo transformation to form endometriotic cells that experience periodic shedding and cause neonatal uterine bleeding or retrograde bleeding [12,15].

### 4.5. Genetic/Epigenetic Theory

The genetic/epigenetic theory of endometriosis posits that the disease is influenced by a combination of inherited genetic predispositions and acquired epigenetic changes in the original cell, for example, an endometrial cell, a stem cell, or a bone marrow cell with inherited genetic and epigenetic defects. These factors contribute to the development and progression of endometriotic lesions, impacting cell behavior, hormonal responses, and treatment outcomes. Understanding this theory provides other insights into the complex pathogenesis of endometriosis [13,16].

## 5. Cellular Mechanisms

**Figure 3:**
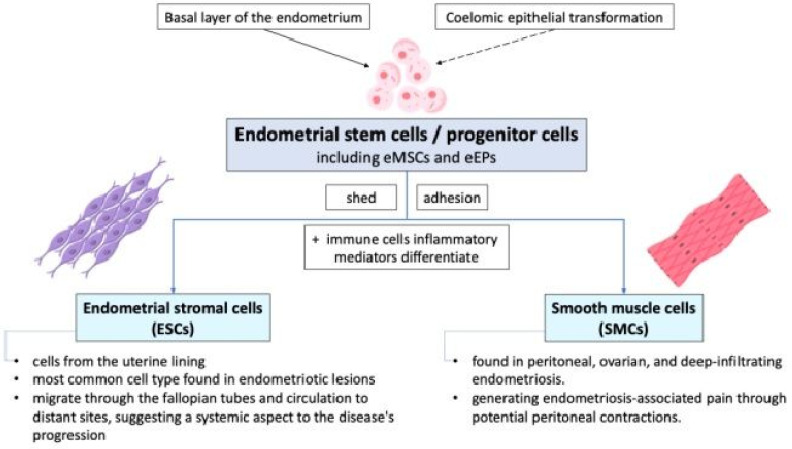
The cellular mechanisms of endometriosis show that endometrial stem cells shed during retrograde menstruation may adhere to peritoneal surfaces due to altered integrin profiles. eMSCs: endometrial mesenchymal stem cells; eEPs: endometrial epithelial progenitors.

Endometrial stem cells or progenitor cells, including endometrial mesenchymal stem cells (eMSCs) and endometrial epithelial progenitors (eEPs), shed during retrograde menstruation and may adhere to peritoneal surfaces due to altered integrin profiles in endometrial stromal cells (ESCs) and smooth muscle cells (SMCs) from women with endometriosis. These cells differentiate into various cell types and are involved in the initiation and sustenance of ectopic endometrial growths. They may originate from the basal layer of the endometrium and can be identified by specific markers such as CD140b+, CD146+, SUSD2+ eMSCs, N-cadherin+, and SP cells [2,6,17,18,19]. ESCs or SMCs have the potential to differentiate into various cell types under the influence of altered peritoneal environments characterized by immune cells and inflammatory mediators. The exact origin and differentiation pathways of these stem/progenitor cells, whether from basal endometrial layers or coelomic epithelial transformation, remain areas of active research. The pathophysiology of endometriosis involves the complex interaction between endometrial stem/progenitor cells, ESCs, and SMCs, as shown in Figure 3.

While retrograde menstruation provides a mechanism for endometrial cells to enter the pelvic cavity, it is the inherent properties and interactions of these cells that facilitate their adhesion, proliferation, and differentiation into sporadic endometriotic lesions [12]. As the cells of the most common cell type found in endometriotic lesions, ESCs coming from the uterine lining play a crucial role in the development and maintenance of these lesions. They can migrate through the fallopian tubes and human circulation to distant sites, suggesting a systemic aspect to the disease’s progression [11,12]. SMCs, which are found in peritoneal, ovarian, and deep-infiltrating endometriosis, may derive from basal stem cells or reactivated coelomic epithelial cells. They are involved in generating endometriosis-associated pain through potential peritoneal contractions.

## 6. Molecular Mechanisms

Retrograde menstruation, while common, is insufficient alone to cause endometriotic lesions. The presence of abnormal stem cells in menstrual fluid, which have heightened implantation and angiogenic capabilities, contributes to the formation of ectopic tissue lesions and provokes localized inflammatory response and immune dysregulation [2,12,20,21].

### 6.1. Adhesion and Invasion

**Figure 4:**
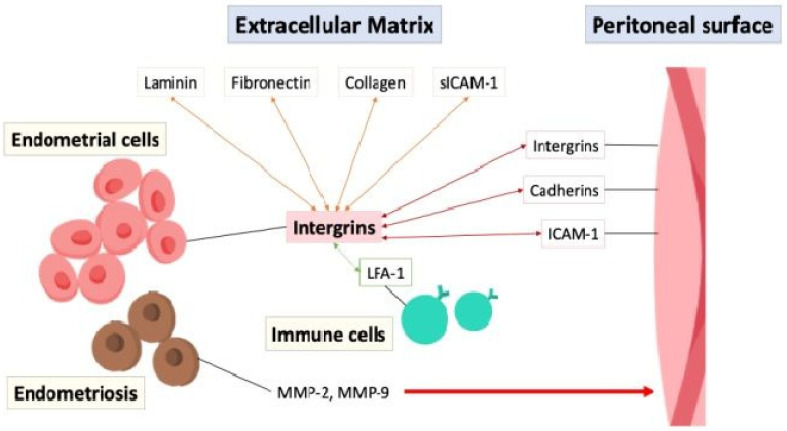
The interaction of cell adhesion molecules (double arrow) between the endometrial cells, extracellular matrix, peritoneal surface, and immune cells. ICAM-1: intercellular adhesion molecule-1; LFA-1: leukocyte function-associated antigen-1; MMP: matrix metalloproteinases; sICAM-1: soluble intercellular adhesion molecule-1.

The adhesion of endometrial cells to the peritoneal surface is possibly mediated through interactions between cell adhesion molecules such as integrins, cadherins, and intercellular adhesion molecule-1 (ICAM-1), with integrins acting on endometrial cells to bind to extracellular matrix (ECM) proteins like laminin, fibronectin, and collagen, facilitating cell adhesion and migration [22]. Soluble intercellular adhesion molecule-1 (sICAM-1) is a soluble form of transmembrane glycoproteins in the extracellular environment and interacts with integrins and other adhesion molecules, particularly the leukocyte function-associated antigen-1 (LFA-1) integrin on immune cells to facilitate cell–cell adhesion and immune responses [23]. The aberrant expression of adhesion molecules, inflammation, or immune dysfunction in the peritoneal cavity can disrupt the balance between cell adhesion and invasion, contributing to the establishment and progression of endometriotic lesions, highlighting their importance in the disease process [24,25]. Furthermore, the endometrial cells in endometriosis secrete matrix metalloproteinases (MMPs), particularly MMP-2 and MMP-9, which act as enzymes that degrade the ECM and remodeling tissue, allowing for penetration and infiltration, increasing invasiveness into the surrounding tissues [26,27,28]. Figure 4 depicts the interaction of cell adhesion molecules between the endometrial cells, extracellular matrix, peritoneal surface, and immune cells.

### 6.2. Proliferation and Apoptosis

#### 6.2.1. Hormones

The mechanism of apoptosis in endometriosis involves a dysregulated balance between pro-apoptotic and anti-apoptotic factors, allowing ectopic endometrial cells to survive and proliferate abnormally. The growth and survival of ectopic endometrial tissues are influenced by hormonal fluctuations. Specifically, the imbalance between estrogen and progesterone signaling is a hallmark of endometriosis [29,30,31]. To support further growth, endometriotic lesions exhibit steroidogenesis, which would increase the expression of aromatase and steroidogenic acute regulatory protein (StAR) while showing decreased expression of 17β-hydroxysteroid dehydrogenase 2 (17β-HSD2), promoting local estrogen production [32,33,34]. Estradiol, the most potent estrogen, promotes the growth and proliferation of endometrial tissues through its combination with estrogen receptors (ERα and ERβ) [20,35]. These two receptors have distinct and sometimes opposing roles in regulating endometrial cell function, inflammation, and apoptosis. In endometriotic lesions, there is an imbalance in ERα and ERβ expression, with ERβ being significantly overexpressed compared to ERα [36,37]. This altered ratio contributes to the survival and proliferation of ectopic endometrial cells, promoting lesion growth by inhibiting TNF-α that induces apoptosis, increasing IL-1β levels which enhance cellular adhesion and proliferation, and facilitating the epithelial–mesenchymal transition [16,38,39,40]. In contrast, ERα is often downregulated in endometriosis, which is typically associated with normal endometrial function and estrogen-driven proliferation. Estrogen–ERα signaling drives the expression of genes such as cyclin D1 and c-Myc, promoting the proliferation of ectopic endometrial cells. ERα activation leads to the upregulation of anti-apoptotic proteins, including Bcl-2, and the downregulation of pro-apoptotic proteins, such as Bax. Additionally, the overexpression of ERβ reduces the effectiveness of ERα-mediated signaling, leading to progesterone resistance and sustained estrogenic activity in ectopic tissues [36,37].

On the other hand, progesterone plays a key role in regulating the menstrual cycle and maintaining endometrial homeostasis. It also has both pro-apoptotic and anti-apoptotic effects depending on the context. Alterations in progesterone signaling, such as progesterone resistance or reduced expression of progesterone receptors (PR-A and PR-B), are observed [20,41] in patients diagnosed with endometriosis. These disruptions are often attributed to epigenetic modifications, such as differential methylation of PR-B, HOX, and GATA transcription-factor genes, which suppress PR-B [38,42]. Moreover, the uncontrolled growth of endometriotic lesions is driven by alterations in hormone-dependent cell cycle molecules such as cyclins and cyclin-dependent kinases, disrupting cell proliferation. For example, the reduction in the progesterone-regulated transcription factor FOXO1A, which is involved in cell cycle control and apoptosis, and the downregulation of the cell cycle regulatory protein ErbB-2 (TOB1), potentially due to elevated interleukin-1β levels, both show significantly reduced expression in endometriotic tissues [43,44].

**Figure 5:**
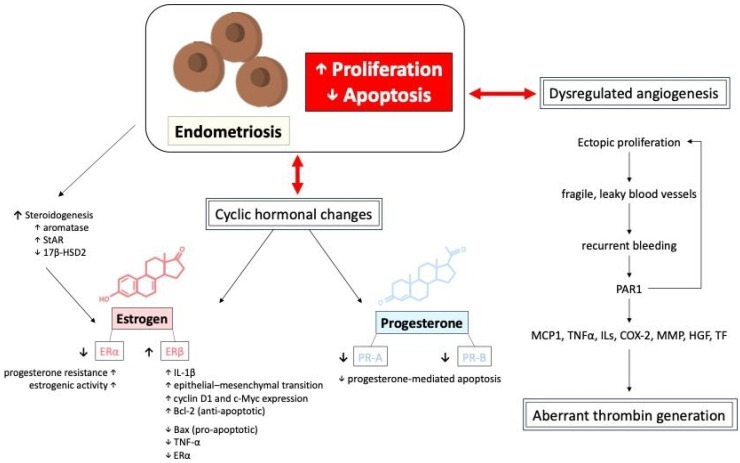
The proliferation, apoptosis, and dysregulated angiogenesis of endometriotic cells, mediated by the interactions of different hormones, cytokines, and tissue factors. 17β-HSD2: 17-beta-hydroxysteroid dehydrogenase 2; COX-2: cyclooxygenase-2; ILs: interleukins; HGF: hepatocyte growth factor; MCP1: monocyte chemoattractant protein-1; MMP: matrix metalloproteinases; TNFα: tissue necrosis factor alpha; PAR1: protease-activated receptor 1; StAR: steroidogenic acute regulatory protein; TF: tissue factor.

Cyclic hormonal changes in ectopic endometrial tissues and dysregulated angiogenesis, resulting in fragile, leaky blood vessels, both contribute to recurrent bleeding episodes in endometriotic lesions, potentially leading to aberrant thrombin generation. Thrombin generation from recurrent bleeding stimulates further endometriotic cell proliferation via protease-activated receptor 1 (PAR1), inducing downstream signaling expression of monocyte chemoattractant protein-1 (MCP1), tissue necrosis factor alpha (TNFα), various interleukins (IL), cyclooxygenase-2 (COX-2), matrix metalloproteinases (MMP), hepatocyte growth factor (HGF), and tissue factor (TF), potentially increasing the risk of thrombotic events [21,27,30,45,46,47,48,49,50]. Moreover, repetitive thrombin production often provokes local inflammation and fibrin aggregation, leading to aggravated adhesion formation and subsequent clinical symptoms in affected patients. The proliferation and apoptosis of endometriotic cells, mediated by the interactions of different hormones, cytokines, and tissue factors, is shown in Figure 5.

#### 6.2.2. Cell Signaling Pathway

Various cell signaling pathways in the endometriotic microenvironment also play a critical role in regulating proliferation, apoptosis, angiogenesis, and immune function. For instance, the estrogen receptors (ERα and ERβ), are transcription factors that modulate gene expression. This binding induces a cascade of signaling pathways, including activation of the PI3K/Akt/mTOR pathway, which promotes cell survival and growth [36,37,51]. The PI3K/Akt pathway is a key signaling cascade involved in cell motility, proliferation, and migration [52,53,54]. The activation of Akt/mTOR signaling can lead to increased cell proliferation and downregulation of autophagy, which inhibits apoptosis [37]. Similarly, aberrant activation of the Wnt/β-catenin pathway or MAPK/ERK pathway may contribute to the invasive and proliferative properties of endometrial cells [32,44,52,55]. In addition, the JAK/STAT pathway is involved in cytokine signaling and immune responses that can be activated by cytokines like PDGF, FGF, and IL-6, as well as angiogenic factors like vascular endothelial growth factor (VEGF) and angiopoietins (ANGs)-1 and 2 [21,28,56,57]. These factors facilitate establishment and vascularization, supporting the growth of endometriotic lesions [58]. Therefore, targeting the JAK/STAT pathway may offer therapeutic opportunities for modulating immune responses and cell behavior in patients who are diagnosed with endometriosis.

#### 6.2.3. Growth Factors

Furthermore, several growth factors, in addition to VEGF, play crucial roles in the pathogenesis of endometriosis [30]. Epidermal growth factor (EGF) stimulates proliferative activity in endometriotic cells, contributing to cell growth and survival within ectopic lesions [38,44,46]. The downregulation of Mitogen inducible gene 6 (MIG6), a negative regulator of EGF signaling, leads to unchecked cell growth. Transforming growth factor-beta (TGF-β) is involved in tissue remodeling, fibrosis, and immune regulation [44,59]. Insulin-like growth factor (IGF) signaling pathways play roles in cell proliferation, survival, and invasion [60]. Platelet-derived growth factor (PDGF) affects cell migration, angiogenesis, and tissue repair, influencing the development and maintenance of lesions. Midkine (MK), a member of the heparin-binding growth factor family, is overexpressed in ectopic endometrium and is implicated in proliferation, migration, angiogenesis, and fibrinolysis [27].

### 6.3. The Role of Immune and Inflammatory Response in Endometriosis

**Figure 6:**
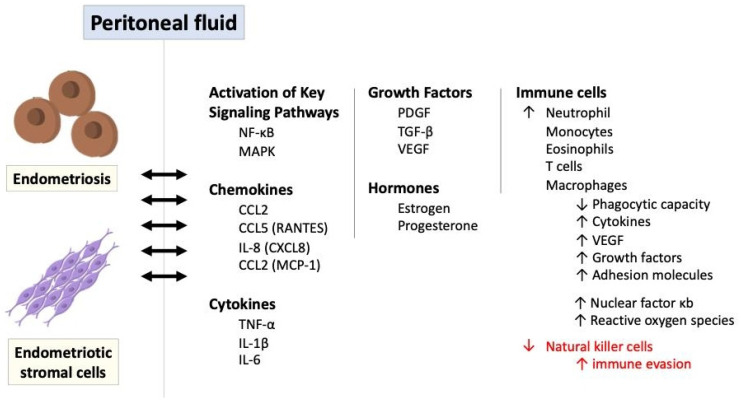
The role of immune and inflammatory response in endometriosis via a variety of mediators and signaling pathways. IL: interleukin; MAPK: mitogen-activated protein kinase; NF-κB: nuclear factor kappa B; PDGF: platelet-derived growth factor; ROS: reactive oxygen species; TGF-β: transforming growth factor-beta; TNF-α: tumor necrosis factor-alpha.

Inflammatory signaling pathways play a significant role in the pathogenesis of endometriosis, contributing to the chronic inflammatory microenvironment, immune dysregulation, and tissue damage associated with the disease [51,61,62]. It leads to the adhesion, invasion, and survival of endometrial cells at ectopic sites, contributing to the pathogenesis of endometriosis. Although dysfunctions in the innate and adaptive immune systems are evident, it is unclear whether this immune dysfunction initiates endometriosis or is only a hallmark of its pathophysiology [61,63]. The immune response in endometriosis primarily occurs in endometriotic stromal cells, endometriotic lesions, and peritoneal fluid within the peritoneal cavity. Figure 6 illustrates the role of immune and inflammatory response in endometriosis via a variety of mediators and signaling pathways.

Ectopic endometriotic cells trigger a localized immune and inflammatory response, producing a variety of cytokines, chemokines, and prostaglandins. For instance, through the expression of chemokines such as interleukin-8 (also known as CXCL8) and CCL2 (referred to as monocyte chemoattractant protein 1 and small inducible cytokine A2) stimulated by endometriotic stromal cells or the production of CCL2 and CCL5 (also known as RANTES-regulated upon activation, normal T cell expressed and secreted) induced by endometriotic lesions, the key immune cells including monocytes, macrophages, neutrophils, T cells, and eosinophils are attracted to the peritoneal fluid [26,46,64,65,66]. These immune cells further secrete inflammatory mediators like platelet-derived growth factor (PDGF) and transforming growth factor-beta (TGF-β), which influence and augment the immune responses in endometriosis. These pro-inflammatory cytokines including TNFα and IL-1 also stimulate leptin, primarily known as the protein released by adipose tissue, and enhance ectopic cell proliferation [67,68].

Additionally, as antigen-presenting cells to activate T cells, macrophages in the peritoneal fluid exhibit reduced phagocytic capacity but heightened production of proinflammatory cytokines such as tumor necrosis factor alpha (TNF-α), interleukin-1β, and interleukin-6, as well as proangiogenic factors like vascular endothelial growth factor (VEGF) with various growth factors and adhesion molecules [3,64,69]. This cytokine production is further amplified by the overexpression of nuclear factor κB (NK-κB) in peritoneal macrophages and endometriotic stromal cells, along with the generation of reactive oxygen species (ROS) and activation of mitogen-activated protein kinase (MAPK) signaling pathways [20,27,48,50,52,70].

The interplay of hormonal and immunological factors, including increased estradiol levels driven by elevated prostaglandin E2 and local aromatase upregulation, underscores the complexity of endometriosis pathogenesis, highlighting the essential roles of cellular adhesion, proliferation, and steroidogenesis. Prostaglandins, particularly prostaglandin E2 (PGE2), and interleukin-17 production by type 17 helper T cells (Th17) are also elevated in the peritoneal fluid of women with endometriosis, further contributing to chronic inflammation [34,71]. On the contrary, natural killer (NK) cell activity is diminished in women with endometriosis, potentially allowing endometrial cells to evade immune detection [26,27]. Since the activity of NK cells is reduced for some reason, their ability to clear the ectopic endometriotic cells is impaired so that the foreign tissues can maintain their growth and replication for a considerable time [72,73,74,75].

### 6.4. Epigenetic Regulation and Gene Expression

Epigenetic mechanisms play a crucial role in the development and progression of endometriosis, influencing gene expression without altering the underlying DNA sequence. These mechanisms include DNA methylation, histone modification, and non-coding RNAs, all of which can impact the pathophysiology of endometriosis. The epigenome can also be influenced by environmental factors, including social behavior, metabolism, and nutritional deficiencies [61,64,76,77].

Aberrant DNA methylation in endometrial tissues can result in the silencing or activation of genes involved in inflammation, cell proliferation, and apoptosis, contributing to the survival and implantation of endometrial-like tissue outside the uterus [5,35,78]. The endometrial tissues can alter gene expression, including that of HOX genes, which play a critical role in endometrial development and receptivity. The dysregulation of HOX genes, along with genes involved in inflammation, cell proliferation, and apoptosis, contributes to the survival, implantation, and persistence of endometrial-like tissue outside the uterus, as seen in endometriosis [79,80].

**Figure 7:**
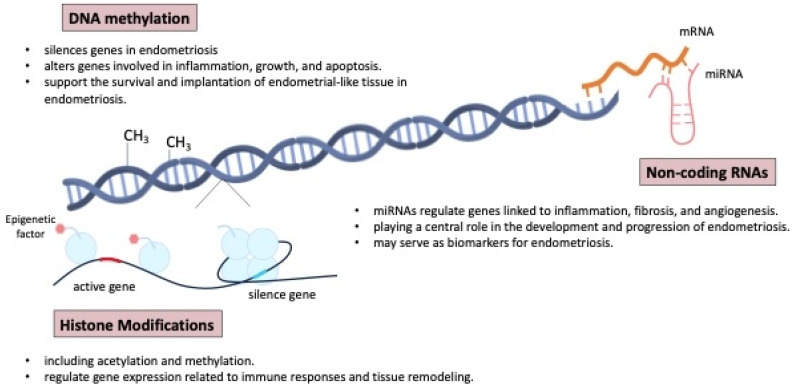
The mechanisms of epigenetic regulation involved in the pathogenesis and development of endometriosis. miRNAs: microRNAs.

Figure 7 shows the mechanisms of epigenetic regulation involved in the pathogenesis and development of endometriosis. Histone modifications, such as acetylation and methylation, also play a key role in regulating gene expression related to immune responses and tissue remodeling in endometriosis [5]. Furthermore, non-coding RNAs, particularly microRNAs (miRNA), have been shown to regulate the expression of genes involved in inflammation, fibrosis, and angiogenesis, all of which are central to the pathogenesis of endometriosis [44,81,82,83,84,85,86,87,88]. Some non-invasive biomarkers, proteomics, genomics, and miRNA microarrays have gained attention and may aid diagnosis, but larger studies and deeper pathophysiologic insights are needed [27,78].

A gene expression analysis revealed altered profiles in endometrial tissues from women with endometriosis, highlighting progesterone resistance and potential susceptibility genes, as well as the involvement of genes like OCT4, osteopontin, prominin-1 (CD133), and stemness-related genes in the disease’s pathogenesis [61,64,89,90]. The expression patterns of these genes in endometrial and endometriotic tissues provide insights into the molecular mechanisms underlying the disease.

This pathway highlights the intricate interactions between adhesion, proliferation, invasion, angiogenesis, and immune responses in the pathogenesis of endometriosis. Dysregulation of these processes contributes to the establishment and progression of endometriotic lesions in the pelvic cavity.

## 7. Symptoms

Endometriosis-related symptoms can significantly impact a woman’s overall health, as well as her mental and social well-being, leading to a marked decline in quality of life [3,91]. The condition is primarily characterized by chronic pain and infertility, which are the most common and debilitating symptoms [92]. Pelvic pain, often described as severe and cramp-like, is the hallmark of endometriosis and typically occurs during menstruation, a condition known as dysmenorrhea. However, pain can also occur at other times in the menstrual cycle, including during intercourse (dyspareunia), bowel movements, or urination, especially when endometrial-like tissue is located on the bladder or bowel [4,9,81]. The severity of pain can vary greatly among individuals, with some experiencing debilitating discomfort that interferes with daily activities. In addition to pain, infertility is a significant issue for many individuals with endometriosis. The condition is associated with anatomical changes such as adhesions, cysts, and distorted pelvic anatomy, which can interfere with ovulation, egg transport, and implantation [81,93,94]. It is estimated that 30–50% of women with endometriosis experience infertility, making it one of the leading causes of female infertility [95,96]. The interplay between pain and infertility often impacts not only physical well-being but also emotional health, as individuals navigate the challenges of managing both chronic pain and difficulty conceiving [97,98].

## 8. Diagnosis of Endometriosis

Historically, diagnosing endometriosis was challenging, often delayed for years due to limited awareness and reliance on invasive methods, where lesions were visually confirmed during open surgery. Clinical history, including symptoms like chronic pelvic pain, dysmenorrhea, and infertility, played a key role, but the overlap with other conditions made diagnosis uncertain. The advent of laparoscopy in the late 20th century marked a major advance, allowing minimally invasive visualization and biopsy for confirmation, establishing it as the gold standard [4,10,92,99,100]. Nowadays, although biochemical markers are not very useful, the introduction of imaging techniques such as transvaginal ultrasound (TVUS) and MRI has shifted the focus towards non-invasive diagnostics, reducing the need for surgical intervention and enabling earlier, more accurate identification [1,10,101].

Guidelines from major organizations, including the European Society of Human Reproduction and Embryology (ESHRE), the American College of Obstetricians and Gynecologists (ACOG), the National Institute for Health and Care Excellence (NICE), and the World Endometriosis Society (WES), offer nuanced approaches to its diagnosis. Table 1 lists the current guidelines for the diagnosis of endometriosis.

### 8.1. ESHRE Guidelines [102]

The ESHRE guidelines emphasize a comprehensive approach to diagnosis. Clinical symptoms like chronic pelvic pain, dysmenorrhea, dyspareunia, infertility, and cyclic bowel or bladder symptoms are key indicators. However, ESHRE prioritizes imaging studies, recommending TVUS as the first-line diagnostic tool for identifying ovarian endometriomas or DIE, with MRI as a secondary option for complex cases. Laparoscopy remains the gold standard for definitive diagnosis, particularly when histological confirmation is sought, but ESHRE advises against routine reliance on surgical methods if imaging and symptoms strongly suggest the disease.

### 8.2. ACOG Guidelines [103]

The ACOG guidelines similarly highlight the importance of clinical symptoms and imaging, with a focus on TVUS for detecting ovarian endometriomas. ACOG recognizes that many cases can be effectively managed based on a presumed diagnosis without the need for invasive procedures. While laparoscopy is still considered the definitive diagnostic method, it is recommended only when imaging results are inconclusive, symptoms persist despite treatment, or surgical management is planned.

### 8.3. NICE Guidelines [104]

The NICE guidelines adopt a more conservative stance, discouraging routine use of laparoscopy for diagnosis unless imaging or symptoms remain unclear despite treatment. The guidelines encourage clinicians to suspect endometriosis in women presenting with chronic pelvic pain, dysmenorrhea unresponsive to treatment, or subfertility, urging early symptom management to avoid delays in care. NICE also emphasizes non-invasive methods, recommending TVUS as the primary imaging modality and reserving MRI for ambiguous findings.

### 8.4. WES Guidelines [105]

The WES guidelines align with others in prioritizing non-invasive diagnostic approaches. They recommend TVUS and MRI as primary tools for detecting ovarian and deep infiltrating lesions, with laparoscopy reserved for cases where imaging is inconclusive or surgery is planned. WES stresses the importance of minimizing diagnostic delays and highlights the role of clinical evaluation and imaging in facilitating earlier management and improving patient outcomes.

## 9. Clinical Treatment of Endometriosis

Endometriosis is a multifaceted condition that necessitates an integrative and personalized approach to management, targeting pain relief, preservation of fertility, and reduction in disease progression. The clinical management of endometriosis involves medical, surgical, and adjunctive therapies tailored to individual patient needs. This section explores these treatment modalities in detail, focusing on their mechanisms, efficacy, and potential advancements. Another important issue is endometriosis-associated pain. Produced by activated endometriotic glands, PGE2 promotes inflammation, pain, and cell proliferation in endometriosis through the activation of specific prostaglandin receptors [106]. Therefore, inhibition of the prostaglandin pathway has been explored as a potential therapeutic target for managing inflammation and symptoms associated with endometriosis [107].

### 9.1. Medical Management

#### 9.1.1. Hormonal Therapies

Hormonal therapies aim to suppress ovulation and menstruation, reducing the ectopic endometrial tissue’s exposure to cyclic hormonal changes. This approach alleviates pain and slows disease progression by creating a hypoestrogenic or pseudopregnancy state. Common hormonal treatments include the following:

- Combined Oral Contraceptives (COCs): COCs are a first-line treatment for endometriosis-associated pain. By suppressing ovulation and inducing decidualization and atrophy of endometrial tissue, COCs reduce pain severity [108]. Continuous regimens are often preferred to eliminate menstruation altogether.
- Progestins: Progestin-only therapies (e.g., medroxyprogesterone acetate and norethindrone acetate) act by inducing endometrial atrophy and suppressing gonadotropin secretion [109]. Dienogest, a novel synthetic progesterone, has shown good therapeutic effects in alleviating endometriosis-associated pain and reducing the progression and recurrence of endometriosis [110]. On the other hand, levonorgestrel-releasing intrauterine systems (LNG-IUSs) provide targeted progestin delivery, reducing systemic side effects while alleviating dysmenorrhea and pelvic pain [111].
- Gonadotropin-Releasing Hormone (GnRH) Agonists and Antagonists: As a modulator of the hypothalamus–pituitary–gonadol axis, GnRH regulates the secretion of gonadotropins (FSH and LH) from the pituitary gland, which in turn control ovarian hormone production and affect the growth of ectopic endometrial tissues. GnRH agonists (e.g., leuprolide acetate) initially induce a flare-up of gonadotropin secretion, followed by the downregulation of pituitary receptors and the subsequent suppression of ovarian estrogen production [112]. Thus, GnRH agonists are used in the treatment of endometriosis to suppress ovarian function and reduce estrogen levels, thereby alleviating symptoms and inhibiting the growth of endometriotic lesions [113]. In contrast, GnRH antagonists (e.g., Elagolix) directly inhibit gonadotropin release, rapidly reducing estrogen levels without an initial flare-up [114]. These therapies are effective for refractory cases but may require add-back hormonal therapy to mitigate hypoestrogenic side effects such as bone loss and vasomotor symptoms.
- Aromatase Inhibitors: Aromatase inhibitors (e.g., letrozole, anastrozole) inhibit estrogen synthesis within ectopic endometrial tissue and the ovary [115]. These are used in combination with progestins or GnRH analogs for severe or refractory cases.

#### 9.1.2. Non-Hormonal Pharmacological Options

- Nonsteroidal Anti-Inflammatory Drugs (NSAIDs): NSAIDs (e.g., ibuprofen and naproxen) are widely used for managing dysmenorrhea and pelvic pain [116]. By inhibiting cyclooxygenase (COX) enzymes, NSAIDs reduce prostaglandin synthesis, alleviating inflammation and nociceptive signaling [117].
- Neuromodulators: For chronic and neuropathic pain, medications such as gabapentin or amitriptyline may be employed as adjunctive therapies [118].

#### 9.1.3. Emerging Medical Therapies

- Selective Progesterone Receptor Modulators (SPRMs): Agents such as ulipristal acetate are being investigated for their ability to modulate progesterone activity selectively, reducing lesion growth while preserving endometrial receptivity [119]. However, they are not used anymore because of the possible side effect of fulminant hepatitis.
- Immunomodulators: Therapies targeting inflammatory and immune pathways, such as tumor necrosis factor-alpha (TNF-α) inhibitors or interleukin antagonists, are in early-stage research for treating endometriosis [120].

Table 2 is a brief summary of clinical medical treatments for endometriosis.

### 9.2. Surgical Interventions

Surgery is indicated for cases of severe pain unresponsive to medical treatment, suspected malignancy, or infertility. The surgical goals are to excise or ablate endometriotic lesions, restore normal anatomy, and alleviate pain [109].

#### 9.2.1. Laparoscopy

Laparoscopy is the gold standard for diagnosing and treating endometriosis. The procedure involves the following:

- Excision: Complete removal of endometriotic lesions is preferred over ablation to ensure thorough eradication of disease tissue and reduce recurrence [121].
- Adhesiolysis: Surgical removal of adhesions restores pelvic anatomy and improves organ function.
- Ovarian Cystectomy: Removal of endometriomas is recommended, especially in symptomatic cases, to relieve pain and improve fertility outcomes [122].

#### 9.2.2. Laparotomy

Reserved for complex or extensive disease, laparotomy involves open abdominal surgery. It is less commonly performed due to its invasiveness and longer recovery time compared to laparoscopy.

#### 9.2.3. Post-Surgical Considerations

Recurrence of endometriosis is common, particularly in cases where complete excision is not achieved. Adjuvant hormonal therapy post-surgery can reduce recurrence rates and prolong symptom relief.

### 9.3. Fertility Preservation and Management

Endometriosis-associated infertility requires individualized management based on the extent of disease and patient’s reproductive goals. The following management may be considered for affected women.

- Ovulation Induction and Intrauterine Insemination (IUI): For minimal-to-mild endometriosis, controlled ovarian hyperstimulation with IUI can be effective [123].
- In Vitro Fertilization (IVF): For moderate-to-severe cases, IVF offers higher success rates. Pre-treatment with GnRH agonists for 2–3 months prior to IVF may improve outcomes [124].
- Ovarian Reserve Preservation: Early referral to fertility specialists and consideration of oocyte or embryo cryopreservation are crucial for patients at risk of diminished ovarian reserve due to surgical interventions or disease progression [125].

### 9.4. Adjunctive and Alternative Therapies

- Lifestyle Modifications: Regular exercise, a low-inflammatory diet, and stress management techniques may complement conventional treatments [126].
- Acupuncture and Physical Therapy: Evidence supports the role of acupuncture and pelvic floor physical therapy in reducing pain and improving quality of life [127].
- Psychological Support: Cognitive behavioral therapy (CBT) and counseling are beneficial for managing the emotional and psychological burden of chronic pain and infertility associated with endometriosis.

### 9.5. Future Perspectives in Endometriosis Treatment

- Precision Medicine: Advances in genomics and proteomics may enable the development of personalized therapeutic approaches targeting specific molecular pathways involved in endometriosis.
- Gene Editing and Stem Cell Therapy: Experimental therapies exploring gene-editing tools like CRISPR and stem cell-based regenerative approaches hold promise for future treatments.
- Non-Invasive Diagnostics: Liquid biopsy and biomarker-based diagnostics are under development to facilitate early detection and monitoring of treatment response, reducing reliance on invasive surgical procedures.

In summary, the clinical treatment of endometriosis requires a multidisciplinary approach that integrates medical, surgical, and supportive care. Advances in molecular biology and immunology are paving the way for innovative therapies, promising improved outcomes for individuals affected by this challenging condition.

## 10. Discussion

Endometriosis is a complex, multifactorial disorder characterized by the growth of endometrial-like tissue outside the uterine cavity. The intricacies of its molecular and cellular mechanisms highlight the challenges in fully understanding the disease and developing effective therapeutic strategies. This discussion synthesizes the primary theories of pathophysiology, cellular mechanisms, and molecular pathways involved in the disease, emphasizing the interplay of genetic, immunologic, and hormonal factors.

The pathogenesis of endometriosis involves multiple overlapping theories, none of which fully explain the heterogeneity of the disease. Retrograde menstruation, the most widely known hypothesis, provides a logical mechanism for the dissemination of endometrial cells to ectopic sites [106]. However, its inability to account for cases of endometriosis in non-menstruating individuals, males, or distant anatomical locations necessitates complementary explanations [107]. The vascular and lymphatic dissemination hypothesis further supports the notion of systemic spread, while the coelomic metaplasia theory extends the understanding of ectopic lesion formation, particularly in patients with Müllerian anomalies or postmenopausal women [108]. Genetic and epigenetic influences are emerging as crucial contributors to disease susceptibility and progression, highlighting the role of heritable factors and acquired molecular modifications in shaping cellular behaviors [109].

Endometrial stem cells and progenitors, such as eMSCs and eEPs, are critical in the establishment of endometriotic lesions. Their unique capacity for adhesion, proliferation, and differentiation in ectopic environments distinguishes them as central players in disease progression [113]. The altered integrin and cadherin profiles in these cells enhance their ability to adhere to peritoneal surfaces, while the influence of the inflammatory microenvironment further supports their survival and growth. Notably, ESCs and SMCs contribute to lesion heterogeneity, with their potential origins spanning basal endometrial layers and reactivated coelomic epithelium [119]. Understanding the lineage and differentiation of these cells remains a critical area of research, with implications for diagnostic and therapeutic approaches.

The molecular underpinnings of endometriosis are marked by dysregulated processes of adhesion, invasion, proliferation, and apoptosis. Aberrant expression of adhesion molecules such as integrins and ICAM-1 facilitates ectopic implantation, while the secretion of MMPs by endometrial cells promotes extracellular matrix degradation and invasion [111]. Hormonal imbalances, particularly elevated local estradiol levels and progesterone resistance, further exacerbate disease progression. The overexpression of ERβ and suppression of PR-B are hallmarks of endometriotic lesions, mediated by epigenetic modifications and cytokine signaling [115]. Pathways such as PI3K/Akt, Wnt/β-catenin, and JAK/STAT are implicated in cellular proliferation, immune modulation, and angiogenesis, reinforcing the multifaceted nature of disease mechanisms [109]. Recurrent bleeding within lesions and thrombin generation contribute to chronic inflammation, adhesion formation, and cyclical pain, exemplifying the systemic impact of local pathology.

Endometriosis is increasingly recognized as an inflammatory disease, with dysregulated immune responses playing a pivotal role. Peritoneal macrophages exhibit impaired phagocytic function but heightened cytokine production, creating a pro-inflammatory milieu [121]. Elevated levels of TNF-α, IL-1β, and IL-6, along with prostaglandins like PGE2, perpetuate inflammation and pain [108]. Diminished NK cell activity enables ectopic cells to evade immune surveillance, while the overactivation of NF-κB and ROS generation further exacerbates tissue damage. These findings underscore the importance of targeting inflammatory pathways in therapeutic strategies.

The elucidation of molecular pathways has paved the way for targeted therapies in endometriosis. GnRH agonists effectively suppress estrogen production, mitigating symptomatology and lesion growth [111]. Emerging approaches aim to modulate key signaling cascades, such as PI3K/Akt and JAK/STAT, or inhibit prostaglandin and cytokine activity to address inflammation and pain [109]. Novel interventions targeting specific growth factors like VEGF, TGF-β, and IGF hold promise in disrupting angiogenesis and lesion maintenance [119].

The progression of endometriosis is influenced by molecular and epigenetic mechanisms that regulate endometriotic cell adhesion, invasion, and survival. Epithelial–mesenchymal transition (EMT) plays a critical role in early-stage lesion establishment, where endometrial epithelial cells acquire a mesenchymal phenotype, enhancing their migratory and invasive properties. Aberrant DNA methylation and histone modifications in genes related to adhesion molecules (e.g., integrins and cadherins) contribute to increased attachment of ectopic endometrial cells to the peritoneal surface. Additionally, microRNAs (miRNAs) dysregulate signaling pathways such as TGF-β, Wnt/β-catenin, and PI3K/Akt, promoting cell proliferation and invasion. As the disease advances to moderate and severe stages, chronic inflammation and angiogenesis play a crucial role, with elevated levels of pro-inflammatory cytokines (e.g., IL-6, TNF-α) and vascular endothelial growth factor (VEGF) sustaining lesion growth and fibrosis. These molecular alterations collectively drive the progression from isolated peritoneal lesions to deeply infiltrating and fibrotic disease, highlighting the complexity of endometriosis pathogenesis [44,81,82,83,84,85,86,87,88].

Emerging clinical evidence suggests a potential role of thyroid hormones in the pathogenesis and progression of endometriosis. Studies have demonstrated that thyroid-stimulating hormone (TSH) acts as a proliferative and pro-oxidative agent on endometrial cells in both endometriosis patients and healthy controls. Additionally, the thyroid hormones triiodothyronine (T3) and thyroxine (T4) specifically enhance proliferation and reactive oxygen species (ROS) production in ectopic endometrial cells. These findings indicate that thyroid hormones may contribute to the establishment and exacerbation of endometriotic lesions through mechanisms involving increased cell proliferation and oxidative stress [128].

Endometriosis-associated ovarian cancer is classified into two main types: those with transitional elements, including atypical endometriosis and borderline tumors, termed endometriosis-correlated or incidental benign endometriosis, and cases of ovarian cancer that are not linked to endometriosis. The former group represents a continuum of malignant transformation, where endometriosis progresses through atypical cellular changes and borderline lesions before developing into invasive carcinoma, typically endometrioid or clear cell carcinoma. In contrast, ovarian cancers without endometriosis association arise independently, often with distinct molecular and histopathological characteristics [129]. Overall, the risk of malignant transformation of endometriosis is relatively low, estimated at 0.7–1.0%, but it is significantly increased in cases of long-standing, atypical, or deeply infiltrating endometriosis (DIE). According to a cohort study of 78,893 women with endometriosis, ovarian cancer risk was higher among women with endometriosis compared with women without endometriosis (adjusted hazard ratio [aHR], 4.20 [95% CI, 3.59–4.91]), and risk of type I (endometrioid, clear cell, mucinous, and low-grade serous) ovarian cancer was especially high (aHR, 7.48 [95% CI, 5.80–9.65]). Ovarian cancer risk was highest in women with DIE and/or ovarian endometriomas for all ovarian cancers (aHR, 9.66 [95% CI, 7.77–12.00]), type I ovarian cancer (aHR, 18.96 [95% CI, 13.78–26.08]), and type II (high-grade serous) ovarian cancer (aHR, 3.72 [95% CI, 2.31–5.98]) [130]. In conclusion, ovarian cancer risk was markedly increased among women with ovarian endometriomas and/or DIE. Genetic alterations, chronic inflammation, and hormonal influences are key drivers of this transformation, highlighting the importance of monitoring patients with endometriosis for early detection of malignant changes. This population may benefit from counseling regarding ovarian cancer risk and prevention and could be an important population for targeted screening and prevention studies [130].

This study has limitations. First, the retrieval of target publications is performed from two databases (Ovid Medline and PubMed) rather than from all databases, which in turn may lead to sampling bias. Furthermore, the eligibility of retrieved articles is evaluated using not only objective criteria but also the subjective judgement of two reviewers, which may subsequently result in selection bias.

## 11. Conclusions

In summary, invasion and proliferation in endometriosis involve a complex interplay of molecular pathways, cell adhesion molecules, angiogenesis, and immune responses that collectively contribute to the invasive behavior of endometrial cells and the progression of the disease. The molecular and cellular mechanisms of endometriosis illustrate a disease driven by intricate interactions between genetic, hormonal, and immune factors. Continued research is essential to unravel the interplay between these processes, refine diagnostic tools, and develop innovative therapies. By addressing the root causes of lesion formation and progression, a more comprehensive understanding of endometriosis can translate into improved outcomes for affected individuals.

## Figures and Tables

**Table 1:** Current guidelines for the diagnosis of endometriosis.

Organization	Key Diagnostic Points	Differences
ESHRE	- Emphasizes non-invasive imaging (transvaginal ultrasound, MRI). - Symptoms: chronic pain, infertility.	Recommends reducing reliance on routine laparoscopy for diagnosis.
NICE	- Focuses on early symptom recognition (pelvic pain, dysmenorrhea). - Suggests timely specialist referral.	Highlights management pathways alongside diagnostic recommendations.
ACOG	- Clinical evaluation as a primary tool. - Laparoscopy as the gold standard for confirmation.	Retains laparoscopy as a key diagnostic method, unlike ESHRE’s reduced emphasis on its routine use.
WES	- Promotes unified, evidence-based diagnostic approaches globally.	Collaborates with multiple organizations to create international standards.

**Table 2:** A brief summary of clinical medical treatments for endometriosis.

Medication	Principles of Actions	Possible Side Effects
Combined Oral Contraceptives (COCs)	Suppress ovulation and induce decidualization and atrophy of endometrial tissue	Elevated risk of gall stone and deep vein thrombosis
Progestins	Induce endometrial atrophy and suppress gonadotropin secretion	Irregular uterine bleeding; body weight gain
Gonadotropin-Releasing Hormone (GnRH) Agonists and Antagonists	As a modulator of the hypothalamus–pituitary–gonadol axis, GnRH regulates the secretion of gonadotropins (FSH and LH) from the pituitary gland, which in turn control ovarian hormone production and affect the growth of ectopic endometrial tissues	Menopausal syndrome and osteoporosis
Aromatase Inhibitors	Inhibit estrogen synthesis within ectopic endometrial tissue and the ovary	Increased risk of osteoporosis and bone fracture; hot flashes; myalgia
Nonsteroidal Anti-Inflammatory Drugs (NSAIDs)	Inhibit cyclooxygenase (COX) enzymes and reduce prostaglandin synthesis, alleviating inflammation and nociceptive signaling	Gastrointestinal tract bleeding and ulcer; heartburn
Neuromodulators	May be employed as adjunctive therapies to alleviate chronic and neuropathic pain,	Nausea; headache; blurred vision
Selective Progesterone Receptor Modulators (SPRMs)	Modulate progesterone activity selectively, reducing lesion growth while preserving endometrial receptivity	Fulminant hepatitis
Immunomodulators	Target inflammatory and immune pathways, such as TNF-α inhibitors or interleukin antagonists	Fatigue; diarrhea; nausea
